# Clinical and histopathological characteristics of atrophic pigmented dermatofibrosarcoma protuberans: A retrospective study of 14 cases

**DOI:** 10.1016/j.heliyon.2024.e39271

**Published:** 2024-10-11

**Authors:** Yufei Zhang, Longfei Zhu, Ying Guo, Christopher Cook, Wenqi Ma, Yutong Ran, Xiaoqian Hu, Yumin Xia, Songmei Geng, Yale Liu

**Affiliations:** a Department of Dermatology, The Second Affiliated Hospital of Xi'an Jiaotong University, Shaanxi, China; b Department of Molecular and Cell Biology, University of California, Berkeley, Berkeley, CA, United States

**Keywords:** Atrophic pigmented dermatofibrosarcoma protuberans, Indolent character, Good prognosis

## Abstract

**Background:**

Dermatofibrosarcoma protuberans (DFSP) invades the dermis and subcutaneous tissue. DFSP with both atrophic and pigmentary (AP-DFSP) features is extremely rare and the clinical characteristics remain unknown. Here we aim to characterize the clinical, histopathologic and prognostic features of AP-DFSP.

**Methods:**

Fourteen cases of patients with AP-DFSP were collected from our institution and published online, including four unreported cases and ten published cases. The clinical appearance, immunohistochemical markers, treatment, and prognosis were analyzed to obtain the clinical and histological features.

**Results:**

There were six males and eight females with a mean age of 25 years old. The vast majority of lesions appeared in the trunk (10/14, 71.4 %) and limbs (3/14, 21.4 %), whereas a minority involved the infraorbital area (1/14, 7.2 %). The most typical manifestation was a depressed plaque-like lesion with fuchsia and bluish color. Histologically, AP-DFSP harbored both atrophic and pigmented features, presenting with a thinner dermis and intradermal melanin granules. Immunohistochemically, CD34 and vimentin were positive while S100 was negative in tumor tissues. The Ki67 index was less than 10 %. Thirteen of fourteen patients had complete excision surgery and follow-ups showed no local recurrence or distant metastasis.

**Conclusion:**

Compared to DFSP, AP-DFSP shows more benign clinical and histological features with a good prognosis. Surgical intervention leads to a significant reduction in tumor burden and dramatically increases the likelihood of complete remission.

## Highlights

• Atrophic pigmented dermatofibrosarcoma protuberans (AP-DFSP) is extremely rare and harbors both atrophic and pigmentary features.
• AP-DFSP appears predominantly in young people with a mean age around 25 years old.
• Compared to classical DFSP, AP-DFSP has a much more indolent feature and a good prognosis after surgical excision.

## 1 Introduction

Dermatofibrosarcoma protuberans (DFSP) is a rare, locally aggressive dermal-based sarcoma that makes up around 0.1 % of all malignancies [1]. With incidences ranging from 0.8 to 4.2 in one million people per year, DFSP has a slight male predominance, and frequently affects the trunk and limbs of young to middle-aged people [2,3]. An asymptomatic, slow-growing nodule or plaque is the hallmark of DFSP. DFSP is composed of parallel spindle cells which frequently metastasize along the interval of subcutaneous adipose tissue [4]. Prior research has demonstrated that DFSP is marked by a rearrangement of chromosomes 17 and 22, leading to the formation of the COL1A1-PDGFB fusion gene [5,6]. This fusion gene is in charge of activating platelet derived growth factor receptor beta (PDGFB) in the form of an autocrine-paracrine cycle [7].

Several variants of DFSP have been identified, including pigmented DFSP (Bednar tumor), sclerotic DFSP, myxoid DFSP, atrophic DFSP, fibrosarcomatous DFSP (FS-DFSP), myoid/myofibroblastic DFSP, and DFSP with areas of giant cell fibroblastoma [[8], [9], [10]]. Histologically, DFSP presents as spindle cells with CD34- and vimentin-positive staining that distinguishes it from dermatofibroma. DFSP also shows S100-negative staining, which can be used to distinguish it from nerve tumors and melanoma [11]. The nuclear protein Ki67 promotes ribosomal RNA synthesis and is essential for cell proliferation. In cancer, a Ki67 index of 30 % or more is considered high and correlates with an increased risk of distant metastasis, cancer-specific mortality, and overall death [12]. DFSP always has a Ki67 index >10 % [13]. Pigmented DFSP and atrophic DFSP are rare variants of DFSP, representing less than 5 % and 1.7 % of all cases, respectively [10,14]. However, DFSP with both atrophic and pigmentary (AP-DFSP) features is extremely rare, and because of its atypical presentations, misdiagnosis is common and treatment is delayed [8,14,15]. To date, only ten cases of AP-DFSP have been reported [8,10,[16], [17], [18], [19], [20], [21]]. Therefore, the clinical features of AP-DFSP remain uncharacterized. Here, we analyzed fourteen cases to reveal the clinical, histopathological and prognostic characteristics of AP-DFSP with the goal of improving diagnosis and treatment.

## 2 Materials and methods

### 2.1 New cases

Four unpublished cases of AP-DFSP were retrieved from the Department of Dermatology, the Second Affiliated Hospital of Xi ‘an Jiaotong University, from January 1, 2014 to December 20, 2023. Medical and pathological reports were used to gather clinical and macroscopic features. The referring pathologist, clinician, the patients or their family were contacted directly over the phone for follow-up information. Age, sex, tumor morphology, size, location, causation, time from onset to diagnosis, and treatment were noted for each patient. All participants provided informed written consent, and their relevant medical backgrounds. Follow-up information from AP-DFSP patients, like recurrence or metastasis, was also gathered. The interval between the first operation and the recurrence or metastasis was used to calculate recurrence or metastasis. The follow-up period was measured as the time of the first surgery to the last follow-up.

### 2.2 Hematoxylin-eosin (HE) staining and immunohistochemistry (IHC)

In order to validate the diagnosis of AP-DFSP, HE and IHC staining were examined in all unreported cases. HE staining was carried out according to routine protocols [22]. After deparaffinization and rehydration, sections were stained for 5 min with hematoxylin and then counterstained for 3 min with eosin. Sequentially, 95 % ethanol, absolute ethanol, xylene, and resin mounting media were all applied to the stained sections. The dermal layer thickness was assessed using NDP. view 2. To evaluate changes in dermal thickness, measurements were taken from corresponding normal skin regions at the same anatomical sites, including peri-nevus skin, from three separate control subjects for comparison.

IHC staining was conducted as described previously [23]. Briefly, paraffin sections were deparaffinized and rehydrated, followed by addition of Dual Endogenous Enzyme Block (DAKO, Glostrup, Denmark). Antibodies against the following antigens were used as the primary antibody: CD34 (Catalog # ZM-0046, ZSGB-BIO, China), S100 (Catalog # ZA-0225, ZSGB-BIO, China), Ki67 (Catalog # GMO27, ZSGB-BIO, China), and Vimentin (Catalog #J10615, Ventana Medical Systems, Inc). Polymer-horseradish peroxidase–labeled goat anti-rabbit and anti-mouse IgG (Roche) were applied as the second antibody. The Ki67 index was determined by three independent researchers based on the positive percentage per high-power field (HPF) ( × 400). In each case, the following histological characteristics were assessed: (1) involved tissue (dermis, adipose tissue, muscle), (2) presence of melanin pigmentation, (3) a mitotic rate per 10 HPFs.

### 2.3 Bibliographical search and data extraction

A search was carried out via PubMed and Google Scholar with the following terms: “pigmented” AND “atrophic” AND “dermatofibrosarcoma protuberans”. Eight articles with 10 cases were included after screening [8,10,[16], [17], [18], [19], [20], [21]]. The following information was extracted from each article: clinical features (age, sex, tumor morphology, size, location, causation, time from onset to diagnosis), pathological features (HE-staining, dermal reduction rate, a mitotic rate per 10 HPFs, the expression of CD34, vimentin, S100 and Ki67), treatment methods, and outcomes.

### 2.4 Statistical analysis

Statistical analysis was carried out, using GraphPad Prism, version 9.0 (GraphPad Software, La Jolla, CA). All data are expressed as mean ± standard deviation (SD). The normal dermal thickness corresponding to each case was measured using the healthy skin from three patients with the same age, sex, and location and compared to each diseased patient by three independent researchers. The difference in dermal thickness between normal and diseased sites is reported here as reduced dermal thickness, and the ratio of reduced dermal thickness to normal dermal thickness is reported as the dermal reduction rate. Five low-power fields were examined within each case. The two groups were compared using the two-tailed Student's t-test with *p* < 0.05 deemed as statistically significant.

## 3 Results

### 3.1 Clinical features

**Fig. 1:**
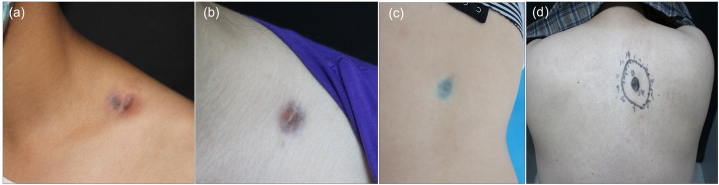
Clinical features of our four AP- DFSP patients. (a–b) Purple pigmented atrophic patch on left supraclavicular fossa of case 1 and 2. (c) Bluish plaque-like lesion on right lower back of case 3. (d) Dark violet atrophic patch on right upper back of case 4. (For interpretation of the references to color in this figure legend, the reader is referred to the Web version of this article.)

The clinical characteristics from 14 patients are presented in Table 1. The ages ranged from seven to 45 years old (mean 25.86 years old). There were six males and eight females. The majority of lesions occurred in the trunk (10/14, 71.4 %), including the back (n = 6), clavicle fossa (n = 2), buttock (n = 1), and shoulder (n = 1). The second most affected area were the extremities (3/14, 21.4 %), including the upper limb (n = 3). The remaining one tumor developed in the left infraorbital region (1/14, 7.2 %). The left side of the body (10/14, 71.4 %) was more common than the right side (4/14, 28.6 %). The patients’ macroscopic features consisted of a depressed plaque-like lesion with purple and bluish color (Fig. 1a–d), with a mean size of 21.38 × 14.85 mm (Table 1). Prior to diagnosis, all lesions had a history of progressive development. The mean time from tumor onset to diagnosis was 8.69 years (range, 2–20 years) among the 13 patients with available data.

**Table 1:** Clinical features of fourteen cases of atrophic pigmented dermatofibrosarcoma protuberans.

Case	Age (year)	Sex	Duration	location	Size (mm)	Tumor morphology	Treatment	Follow-up (months)	Reference
1	9	M	5	Left clavicle fossa	20 × 15	Round purple plaque-like	Wide excision	NED,36	This study
2	33	F	10	Left clavicle fossa	16 × 22	Round purple plaque-like	Wide excision	NED,39	This study
3	20	F	4	Right lower back	18 × 9	Depressed bluish atrophic patch	Wide excision	NED,72	This study
4	44	M	20	Right upper back	10 × 20	Dark violet atrophic patch	Wide excision	NED,21	This study
5	24	F	2	Left infraorbital area	NA	Well-demarcated depressed bluish plaque	Untreated	NA	Chuan *et al*. [16].
6	34	F	15	Left buttock	11 × 12	Pigmented plaque	Wide excision	NA	Taura *et al*. [18].
7	7	F	NA	Left wrist	20 × 40	Pigmented atrophic patch	Wide excision	NED,24	Zhang *et al*. [20].
8	8	F	5	Left forearm	10 × 10	Bluish scleroatrophic plaque	Wide excision	NED,24	Zhang *et al*. [20].
9	7	M	4	Left forearm	5	Purple-red atrophic patch	Wide excision	NA	Xu *et al*. [10].
10	44	M	3	Right back	25	Round bluish plaque-like lesion	Wide excision	NED,44	Xu *et al*. [10].
11	26	M	10	Right back	30 × 25	Pigmented atrophic plaque	Wide excision	NED,7	Bai *et al*. [8].
12	45	M	5	Left shoulder	30 × 20	round erythematous-to-bluish atrophic plaque	Mohs surgery	NA	Li *et al*. [17].
13	33	F	10	Left upper back	16 × 13	Bluish and depressed plaque	Mohs surgery	NED, 12	Lin P *et al*. [21].
14	28	F	20	Left lower back	30 × 14	Bluish and depressed plaque	Wide excision	NA	Gong Y *et al*. [19].

NED, no evidence of disease; NA, not applicable.

### 3.2 Pathological features

**Fig. 2:**
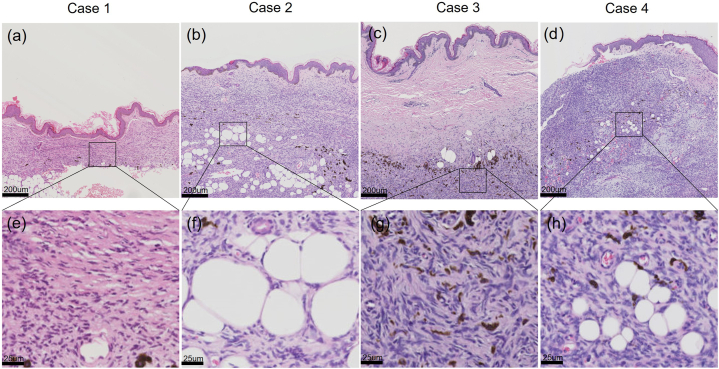
Histological features of AP-DFSP. (a–d) Spindle cell proliferation in the dermis and sporadic pigmented cells of the four cases. Scale bar = 200 μm. (e) Horizontally oriented fascicles in case 1. Scale bar = 25 μm. (f) Subcutaneous infiltration in case 2. Scale bar = 25 μm. (g) The whorled pattern of spindle cells in case 3. Scale bar = 25 μm. (g) Horizontally oriented fascicles and subcutaneous infiltration in case 4. Scale bar = 25 μm.

Histologically, all tumors were dermal-based and presented with a distinctive plaque-like development pattern with pigmented cells scattered among the tumor cells (Fig. 2a–d). The dermal layer was at least 50 % thinner than normal skin tissue (Table 2 and Figs. S1a–b). The dermal thickness of case was reduced by up to 95 % (atrophy from 4437.5 μm to 200.2 μm, *p* < 0.0001). Tumor tissue not only invaded the dermis, but also infiltrated into the subcutaneous adipose tissue (Fig. 2f and h). Some tumor tissue showed monomorphic spindle cells with thin wavy nuclei, arranged in parallel or horizontal fascicles (Fig. 2e). These spindle cells were occasionally organized into helical structures (Fig. 2g). Generally, tumor cells presented with low mitotic activity and minimal cellular atypia (1.80 ± 2.38/10 HPFs) (Table 2).

**Table 2:** Pathological features of fourteen cases of atrophic pigmented dermatofibrosarcoma protuberans.

Case	Dermal reduction rate	#/10HPF	CD34	S100 (Spindle cells)	S100 (pigment-containing cells)	Vimentin	Ki67	Reference
1	80.8 ± 5.6 %	0	+	–	+	+	<5 %	This study
2	85.2 ± 3.1 %	1	+	–	+	+	<5 %	This study
3	76.6 ± 2.5 %	1	+	–	+	+	<10 %	This study
4	95.4 ± 0.9 %	6	+	–	+	+	<5 %	This study
5	NA	NA	NA	NA	+	+	NA	Chuan *et al*. [16].
6	50 %	1	+	–	+	–	NA	Taura *et al*. [18].
7	>50 %	NA	+	–	+	NA	NA	Zhang *et al*. [20].
8	>50 %	NA	+	–	+	NA	NA	Zhang *et al*. [20].
9	NA	NA	+	–	NA	+	<10 %	Xu *et al*. [10].
10	NA	NA	+	–	NA	+	<10 %	Xu *et al*. [10].
11	>50 %	NA	+	–	+	+	NA	Bai *et al*. [8].
12	NA	NA	+	–	+	NA	NA	Li *et al*. [17].
13	NA	NA	+	–	+	+	NA	Lin P *et al*. [21].
14	NA	NA	+	–	NA	NA	NA	Gong Y *et al*. [19].

NA, not applicable.

**Fig. 3:**
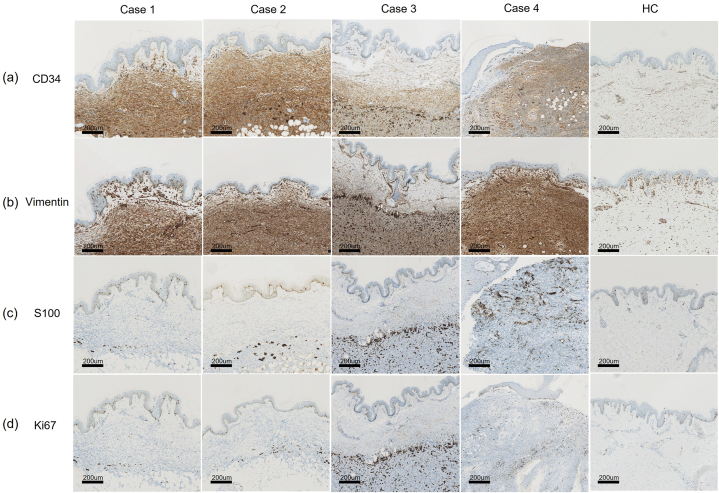
Immunohistochemical characteristics of our four AP-DFSP cases. (a–b) Diffuse and strong expression of CD34 and vimentin in each of the four cases. (c) Positive staining of S100 in pigmented cells and negative staining in tumor in the four cases. (d) Ki67 expression in the four cases. Scale bar in all images = 200 μm.

The IHC results using key diagnostic markers are summarized in Table 2. All tumors displayed diffuse and robust positive CD34 staining and strong positive staining for vimentin (Fig. 3a and b). Further, all spindle cells were negative for S100 protein, except the pigment-containing cells (Fig. 3c). The Ki67 index was less than 10 % in all measurable cases (Fig. 3d).

### 3.3 Treatment and follow-ups

Among 14 patients, 13 patients had surgery without any adjunctive treatment while one patient was left untreated (Table 1). Nine patients with available information were followed up clinically (mean 31 months; range 7–72 months). Either at the last follow-up or according to previous literature reports, none of the patients showed any signs of local recurrence or distant metastasis.

## 4 Discussion

In this study, we show that AP-DFSP is more common in youth. Histologically, AP-DFSP harbors both atrophic and pigmented features, presenting with a thinner dermis and intradermal melanin granules. Immunohistochemically, positive staining for CD34 and vimentin is seen in nearly all AP-DFSP cases, while S100 is negative. The Ki67 index is <10 %, which suggests low mitotic activity. Surgery is a widely used and effective therapy, with follow-up showing little to no recurrence or metastasis. Therefore, our study reveals the benign character of AP-DFSP based on these clinical and histological features.

Compared to classical and atrophic DFSP, AP-DFSP appears predominantly in younger people with a mean age around 25 years [10], with children less than 10 years old accounting for more than 63.6 % of all AP-DFSP cases. Our research team, together with Zhang and others, thus believe that children are more prone to AP-DFSP [20], however more cases will be required to fully verify this. Like classical DFSP, the majority of AP-DFSP lesions (92.8 %) are located on the trunk and extremities. Other areas such as the head region can also be affected but are comparatively rare. The anatomical location shows that the left side is more common in AP-DFSP, which differs from classical DFSP [24]. A stronger neuroimmunomodulation and cell-mediated hypersensitivity in the left side of the body in young people may explain this observation [25], but more research is needed to further investigate this potential anatomical difference.

Generally, the lesions of AP-DFSP are characterized by consistent but slow growth over a long period. The majority of patients have a history of AP-DFSP lasting several years, and tend to wait 10 years or longer before surgery. The patient in case 4 showed no significant deterioration or metastasis for 20 years, while the lesion in case 1 was detected when she was four years old and had not significantly worsened or metastasized until the operation. In light of this, we are more likely to interpret AP-DFSP as having a benign and indolent character.

AP-DFSP shows a typical plaque-like growth pattern with a central depression, and presents with a variety of unevenly distributed bluish and fuchsia colors, which are interpreted as hyperpigmentation [16]. Despite our tendency to believe that AP-DFSP is less severe than typical DFSP, a timely and accurate diagnosis is crucial for patients to have a better prognosis. Clinically, AP-DFSP comprises atrophic lesions, and thus it is frequently mistaken for other diseases featured with atrophy or sclerosis, such as morphea, neurofibroma, resolving panniculitis, lipoatrophy, idiopathic atrophoderma, atrophic scar, atrophic dermatofibroma or steroid atrophy. In addition, AP-DFSP in children also needs to be distinguished from hemangioma and cellular blue nevus [20,26]. One trait that can be used to differentiate AP-DFSP from the aforementioned diseases is a single tough plaque and the absence of spontaneous resolution [20]. AP-DFSP also needs to be differentiated from hemosiderotic fibrous histiocytoma, a subtype of dermatofibroma, which is marked by vascularity and heavy hemosiderin content, instead of the hyperpigmentation. When coloration appears on an atrophic lesion in a young patient, dermatologists should be highly suspicious of AP-DFSP. We found that a purple or blue color is a key characteristic for non-biopsy detection of AP-DFSP in individuals with atrophic lesion on the trunk.

Histologically, like classical DFSP, AP-DFSP is marked by fascicles of spindle cells with parallel, storiform or whorled pattern. Frequently, the tumor cells invade into the subcutaneous adipose tissue. At present, there is a great dispute over the origin of spindle cells. Most scholars believe that it is related to neuroectodermal cell differentiation [27], yet after summarizing the skin lesions of five patients, no nerve cell differentiation was found, and therefore Ding and others proposed that spindle cells come from fibroblasts [28]. The CD34- and vimentin-positive staining further verified that these cells derive from fibroblasts [29,30], yet additional research is needed to investigate their origin. Pigmented DFSP is characterized by the presence of melanin-containing dendritic cells, in addition to the typical histologic features of DFSP [31]. Atrophic DFSP presents as a dermis-based lesion upon histopathological examination. It shows that the local dermis is significantly atrophied, and the thickness of the dermis in the middle of the tumor is reduced to less than 50 % [32]. Therefore, it seems that AP-DFSP harbors features of atrophic DFSP and pigmented DFSP at the same time.

In the tumor tissues of our AP-DFSP patients, cytological atypia is minimal and mitotic activity is pretty low, unlike FS-DFSP. FS-DFSP represents the aggressive form of DFSP with higher risks of local recurrence, metastasis, and death [33,34]. In FS-DFSP, the tumor's FS portion undergoes more mitoses than the DFSP region [33,35,36]. The recurrence (13.7 %) and metastasis (1.1 %) of classical DFSP are common, despite the fact that it also has a low mitotic rate [33]. To some extent, the low mitotic rate of AP-DFSP lesions supports the idea that this condition is more clinically benign [36].

IHC markers not only help clinicians to identify and distinguish DFSP, but also are a signal of tumor progression and prognosis. Research has shown that the regions with FS change in individuals who have lost CD34 immunoreactivity, possibly in line with the development of CD34-negative fibrosarcoma [35,37]. In addition, CD34 expression was also lost in the tumor tissue of a recurrent DFSP patient with myxoid and fibrosarcoma changes [38]. The high CD34-positive staining seen in AP-DFSP suggests that a CD34-associated immune response may mediate immune suppression in AP-DSFP to maintain its benign character [39]. Vimentin is ubiquitously expressed in mesenchymal cells, and largely provides resistance to pressure and stress at the single-cell level [[40], [41], [42]]. By distributing local mechanical stress to other parts of the cell, vimentin creates a hyper-elastic network that increases the cell's stretchability, strength, resilience, and toughness to sustain cell viability. We therefore propose that vimentin overexpression is a response to the stress caused by tissue shrinkage, in addition to demonstrating that AP-DFSP is a source of mesenchymal tissue. Commonly, S100 is used to distinguish DFSP from other tumors, especially neural crest-derived and melanocyte-derived tumors [43,44]. However, current research indicates that S100 is tightly linked to cell invasion and migration [45], which may account for the high immunoreactivity of S100 in the more aggressive FS-DFSP [35]. The lower immunoreactivity of S100 observed in AP-DFSP may correlate with a lower metastasis of AP-DFSP. An alternate independent predictor for recurrence-free survival in multivariate Cox proportional hazards regression analysis is the Ki67 proliferation index, which is useful for facilitating the identification of individuals with DFSP who are at a higher risk of developing disease recurrences [46]. Compared to classical DFSP, the number of Ki67 positive tumor cells was higher in FS- and myxoid DFSP [47]. Our results reveal that the Ki67 index of AP-DFSP is less than 10 %, and therefore suggests lower metastasis. The expression of the above indicators in AP-DFSP needs to be analyzed in larger cohorts, but the information presented above strongly suggests that AP-DFSP is a more benign tumor.

The majority of DFSP (>95 %) patients harbor the imbalanced t (17; 22) or r gene-derived COL1A1-PDGFB fusion gene (17; 22) [10]. COL1A1 encodes the α-chains of type 1 collagen, and PDGFB encodes for the β-chain of platelet-derived growth factor. The latter has powerful mitogenic effects on a variety of cells. As the COL1A1 and PDGFB genes fuse, COL1A1 takes the position of PDGFB's inhibitory regulatory element, enabling the production of high levels of the chimeric COL1A1-PDGFB mRNA. The COL1A1-PDFGB protein undergoes PDGFB cleavage, and the released PDGFB stimulates cells in an autocrine manner to cause malignant transformation [48,49]. Histology and immunohistochemistry are reliable and routine tests for the diagnosis of DFSP. The detection of COL1A1-PDGFB is not necessary in most scenarios. However, it needs to be verified by either fluorescence in situ hybridization or RT-PCR analysis for 5 % of diagnostically challenging cases or to be considered for treatment with imatinib [50,51]. Therefore, COL1A1-PDGFB gene detection in AP-DFSP should be completed to direct both diagnosis and treatment.

Our findings indicate that all AP-DFSP appear to be tiny and occur in relatively simple surgical locations, which further validates its indolent feature. Therefore, we advise early surgical excision and ongoing follow-up. Even with the low metastasis and recurrence of AP-DFSP, surgical margin still needs attention. According to Roses and others, margins of 2 cm or less led to a 41 % recurrence rate, while margins of more than 2 cm had a rate of 24 % [52]. Margins of at least 3 cm encompassing the underlying fascia resulted in the lowest recurrence rate (20 %) [52,53]. Therefore, we recommend using a surgical margin of at least 3 cm and removing superficial fascia to decrease postoperative recurrence. During surgery, fresh frozen sections can assist the surgeon in determining the size of the incision margin [54]. However, in our practice, we found that the tumor tissue of AP-DFSP on frozen sections is not well differentiated from normal tissue, so it is recommended to make routine paraffin sections and add a CD34 marker to distinguish them. In some patients with metastatic disease or inoperable primary or recurring tumors, targeted therapy with imatinib or sunitinib is advised [10,55]. According to clinical trials, imatinib had powerful antitumor effects in advanced dermatofibrosarcoma protuberans harboring t (17; 22) (q22; q13), but care should be taken to monitor for patients who might become imatinib-resistant [49,55].

There are some limitations in our study. Our sample size is small due to the rarity of AP-DFSP, which may cause our results to deviate from the true characteristics of AP-DFSP. Additionally, for previously reported cases, more prolonged follow-up information is not available, therefore prognosis can only be calculated based on the length of follow-up time provided in the prior literature.

In conclusion, our research provides a preliminary description of the clinical and pathological features of AP-DFSP, and demonstrates its benign nature with a good prognosis.

## CRediT authorship contribution statement

**Yufei Zhang:** Writing – review & editing, Visualization, Investigation. **Longfei Zhu:** Writing – review & editing, Validation, Data curation. **Ying Guo:** Software, Resources. **Christopher Cook:** Software, Data curation. **Wenqi Ma:** Validation, Resources. **Yutong Ran:** Validation, Resources. **Xiaoqian Hu:** Resources, Investigation. **Yumin Xia:** Supervision, Methodology. **Songmei Geng:** Supervision, Conceptualization. **Yale Liu:** Supervision, Project administration, Funding acquisition, Data curation, Conceptualization.

## Ethics statement

The study was ethically approved by the ethic committee of the Second Affiliated Hospital of Xi'an Jiaotong University (approval number 2012–003). Written informed consent - according to the principles of the Declaration of Helsinki.

## Data availability statement

The authors have confirmed that the data supporting the findings of this study are available within the article and its supplementary materials.

## Funding declaration

This study was supported by the 10.13039/501100001809National Natural Science Foundation of China (Projects No.81803137) and the Natural Science Foundation of Shaan Xi Province (Projects No. 2023-JC-YB-714).

## Declaration of competing interest

The authors declare that they have no known competing financial interests or personal relationships that could have appeared to influence the work reported in this paper.

## Figures and Tables

**Figure:**
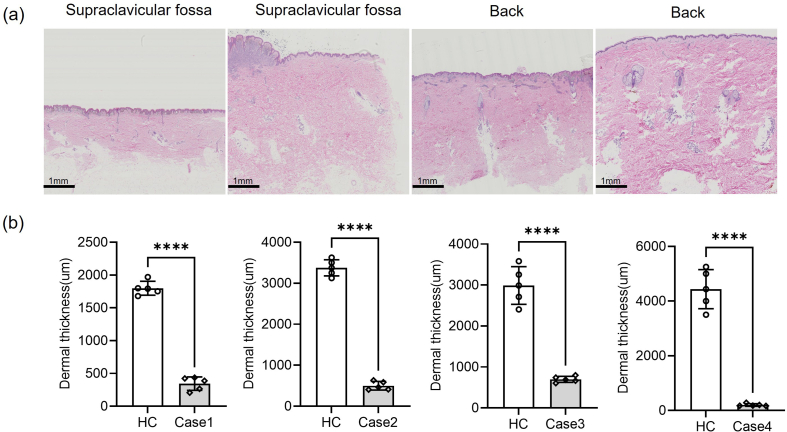
The dermis thickness of corresponding healthy skin (HC) regions at the same anatomical sites. (a) HE staining of HCs. Scale bar = 1 mm (b) Dermal thickness changes between cases and comparative healthy controls. ∗∗∗∗*p* < 0.0001, student's two-tailed *t*-test. Error bars = SD.
